# Exploring Gender Dynamics in ADHD Diagnosis and Treatment Among Adults in a Rural Setting: A Secondary Data Analysis

**DOI:** 10.1192/bjo.2026.11187

**Published:** 2026-06-30

**Authors:** Hezekiah Agboji, Joseph Obanye, Aderonke Agboji

**Affiliations:** 1 GR Baker Memorial Hospital, Quesnel, Canada; 2 University of British Columbia, Vancouver, Canada; 3 University of Northern British Columbia, Quesnel, Canada

## Abstract

**Aims:**

Despite growing recognition of disparities in the diagnosis and treatment of adult attention deficit hyperactive disorder (ADHD), few studies have examined how gender intersects with rural healthcare contexts to shape diagnostic pathways. This gap is particularly concerning given that rural healthcare settings face structural constraints such as limited access to specialized mental health services that may further exacerbate diagnostic inequities, especially for gender-diverse populations. The aims of this study were to explore ADHD subtype distribution among adults attending a rural outpatient clinic, examine demographic and psychosocial correlates of subtype, and assess gender-based variation in these associations using the Gender-Based Analysis Plus (GBA+) framework.

**Methods:**

This is a retrospective cross-sectional study of adults diagnosed with ADHD in an outpatient mental health clinic in rural Northern British Columbia, Canada. ADHD subtypes were classified using two standardized diagnostic assessments: the Diagnostic and Statistical Manual of Mental Disorders, Fifth Edition (DSM–5), and the Diagnostic Interview for ADHD in Adults, version 5 (DIVA–5). Sociodemographic characteristics, psychosocial factors, comorbidities, and medication use were extracted from clinical records. Associations between ADHD subtype and key variables were examined using both descriptive and inferential statistics. Gender-stratified analyses were performed to explore the relationships between ADHD subtype and psychosocial factors, with a focus on treatment patterns and functional indicators relevant to rural health service delivery.

**Results:**

The sample consisted of 660 adults age range between 20 and 75 years (mean age=38 years). The combined presentation was the most prevalent ADHD subtype (67%),followed by the predominantly inattentive presentation (30%); the hyperactive/impulsive presentation was uncommon (3%). ADHD subtype distribution did not differ significantly by gender. The association between ADHD diagnosis subtype and gender was not statistically significant, χ²(4, N=660)=1.10, p=0.894. Employment status also did not differ significantly by ADHD subtype (χ² (2)=4.48, p=0.107), indicating similar employment distributions across combined, inattentive, and hyperactive/impulsive ADHD presentations. In contrast, medication use differed significantly by ADHD subtype (χ² (2)=12.75, p=0.002), with higher rates of pharmacological treatment among individuals with combined presentations.

**Conclusion:**

These findings support adult ADHD guidelines that prioritize comprehensive assessment and individualized care rather than gender-based assumptions. In rural mental health systems, aligning practice with standardized diagnosis, subtype-informed treatment planning, and structured follow-up may strengthen equity, consistency, and continuity in adult ADHD care delivery.

